# Immune-Mediated Change in the Expression of a Sexual Trait Predicts Offspring Survival in the Wild

**DOI:** 10.1371/journal.pone.0025305

**Published:** 2011-10-03

**Authors:** Rémi Chargé, Gabriele Sorci, Yves Hingrat, Frédéric Lacroix, Michel Saint Jalme

**Affiliations:** 1 Muséum national d'Histoire naturelle, Conservation des Espèces, Restauration et Suivi des Populations, Paris, France; 2 Emirates Center for Wildlife Propagation, Province de Boulemane, Missour, Morocco; 3 Université de Bourgogne, Unité Mixte de Recherche, Centre national de la recherche scientifique, Dijon, France; University of Arkanas, United States of America

## Abstract

**Background:**

The “good genes” theory of sexual selection postulates that females choose mates that will improve their offspring's fitness through the inheritance of paternal genes. In spite of the attention that this hypothesis has given rise to, the empirical evidence remains sparse, mostly because of the difficulties of controlling for the many environmental factors that may covary with both the paternal phenotype and offspring fitness. Here, we tested the hypothesis that offspring sired by males of a preferred phenotype should have better survival in an endangered bird, the houbara bustard (*Chlamydotis undulata undulata*).

**Methodology/Principal Findings:**

We tested if natural and experimentally-induced variation in courtship display (following an inflammatory challenge) predicts the survival of offspring. Chicks were produced by artificial insemination of females, ensuring that any effect on survival could only arise from the transfer of paternal genes. One hundred and twenty offspring were equipped with radio transmitters, and their survival monitored in the wild for a year. This allowed assessment of the potential benefits of paternal genes in a natural setting, where birds experience the whole range of environmental hazards. Although natural variation in sire courtship display did not predict offspring survival, sires that withstood the inflammatory insult and maintained their courtship activity sired offspring with the best survival upon release.

**Conclusions:**

This finding is relevant both to enlighten the debate on “good genes” sexual selection and the management of supportive breeding programs.

## Introduction

Identifying the benefits of non random mate choice has been a long lasting focus of sexual selection studies [1]. Indicator models of sexual selection assume that exuberant sexual traits have evolved because they signal the quality of their bearers and the choosy sex can therefore gather a benefit for mating with males with a particular phenotype [2]–[3]. In many organisms, females (usually the choosy sex) can obtain substantial benefits from their mate choice in terms of nuptial gifts, resources transferred with the seminal fluid during the copulation, parental care or more generally resources present in the territory defended by the chosen male [4]. These benefits directly improve female fecundity and confer a selective advantage to choosy females, outweighing the potential cost of choosiness. In many other organisms, however, females do not seem to achieve any direct benefit from their choice, usually because the interaction between sexes is limited to the transfer of genetic material with the male gametes, as for instance in lekking species [5]–[6] or during extra-pair copulations [7]–[8]. Giving the cost of choosiness, one might wonder how female preference has evolved in such systems. In addition to direct benefits, it has been suggested that choosy females can gather indirect benefits in terms of paternal genes [see 9 for a recent review]. Such genetic benefits do not directly improve female fecundity but are supposed to enhance offspring survival. Genetic benefits of mate choice can arise because viability genes (so called good genes) are transmitted from the father to the offspring. These genes with additive effects on fitness provide a benefit whatever the genetic background of the female [10]–[11].

In spite of the enormous interest that indirect sexual selection has given rise to, empirical assessment of the strength of genetic benefits has proven difficult [12]. Some of the best evidence in support to the good genes theory of sexual selection comes from studies on birds and fish. In a seminal paper on a paradigmatic species, Petrie [5] showed that peacocks (*Pavo cristatus*) with the most ornamented train sired offspring with the fastest growth rate and the best survival prospect. Although very suggestive this study and many others that followed could not completely discard the possibility of maternal effects [such as a differential investment of maternal resources depending on male phenotype [13]]. Artificial insemination is a powerful tool that can be used to disentangle genetic and environmental effects due to maternal investment. Artificial insemination has indeed been adopted in recent studies on fish and the results in general support the idea that more ornamented males tend to sire offspring with improved expression of fitness linked traits [14]–[15]. Most of these studies have, however, assessed offspring fitness under laboratory conditions. This is understandable given the difficulty to monitor individual survival under natural conditions. However, this also gives rise to the possibility that the fitness consequences of indirect selection might vanish under the environmental conditions experienced *in natura*.

Here, we take advantage of a houbara bustard (*Chlamydotis undulata undulata*) supportive breeding program, located at the Emirates Center for Wildlife Propagation (ECWP) in Morocco, to examine the contribution of ‘viability’ paternal genes to offspring fitness. The mating system of houbara bustard is based on a so-called exploded lek [16]. In lekking species, the paternal contribution to reproduction is limited to the transfer of genetic material with the gametes [17]. Lekking species are, therefore, ideal systems to investigate the contribution of paternal genes to offspring fitness. Houbara courtship display is characterized by a circular running with the white ornamental feathers on the neck and the head fully erected. Because courtship is energetically costly [16], [18], females may use this courtship display to assess male quality and in doing so choose the healthiest mates. This idea is corroborated by previous work conducted on this and other species showing that the activation of the inflammatory response decreases the intensity of courtship display [19]–[20]. In this study, we took advantage of this previous knowledge to investigate whether i) natural variation in courtship display and ii) the male capacity to withstand an inflammatory insult, as signalled by its courtship display, can be used by females as an indicator of good genes in this endangered species. This aim was achieved by experimentally activating the inflammatory response of males and assessing their courtship activity prior to and following the challenge. Semen of these males was collected and used to inseminate females. Offspring produced by these artificial inseminations were then released into the wild (where they were obviously exposed to a range of ecological risks: starvation, predation, pathogens) and their survival monitored through radio tracking.

We tentatively predict that if courtship display and the capacity to withstand the inflammatory insult are reliable indicators of genetic quality, offspring sired by males with exuberant display and who maintain their courtship activity in spite of the inflammatory challenge should enjoy better survival prospects.

## Results

### Between-year repeatability of courtship display

If courtship display reflects genetic quality (and in the absence of gene-by-environment effects) the expression of the trait should be consistent across years. In agreement with this, we found a highly significant correlation between courtship display across years (2006 and 2007) (r = 0.76, p<0.001) for the 15 sires used here. A linear mixed model of variance, with bird identity declared as a random factor, also showed that among-individual variation in courtship display was significantly higher than the intra-individual (between years) variation (Z = 20.7, p = 0.0191).

### Effect of the LPS injection on courtship display

**Figure 1:**
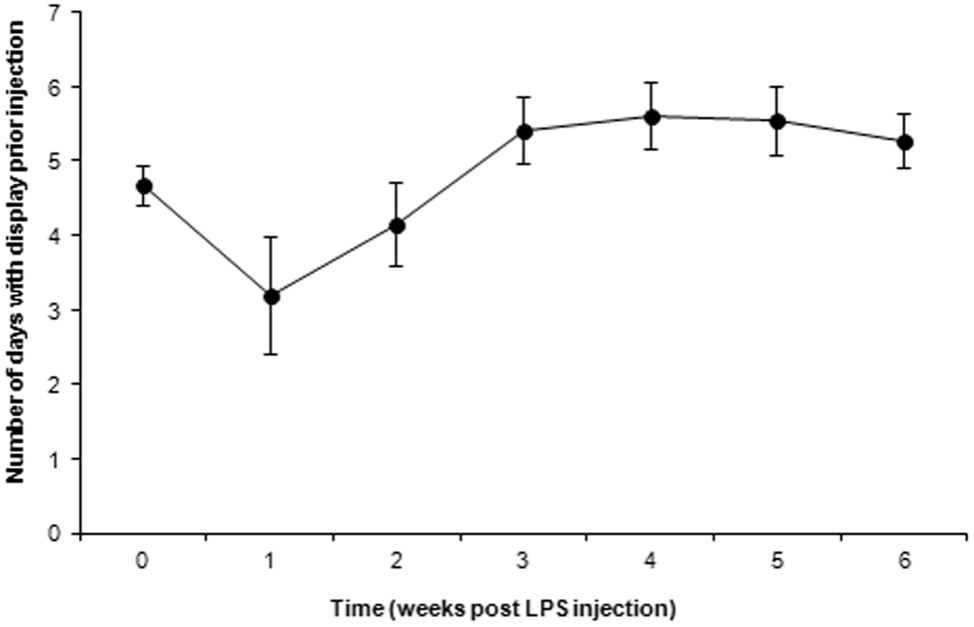
Effect of the LPS injection on the number of days with courtship display in sire houbara bustards in 2007. Values on the y-axis represent the pre-injection values (time 0) and the weekly post-injection values. Bars represent standard errors.

In 2007, LPS-injected sires showed a drop in their courtship display during the week that followed the immune challenge and recovered their initial values at week 3 post-injection (GLMM: time, F_6,105_ = 5.02, p = 0.0001; Fig. 1). Sires differed in their ability to withstand the inflammatory challenge. Among-individual variation in the ability to cope with the immune challenge resulted in a correlation between the percent change in display rate and the pre-injection values (r = 0.642, n = 15, p = 0.0098).

### Offspring survival

**Figure 2:**
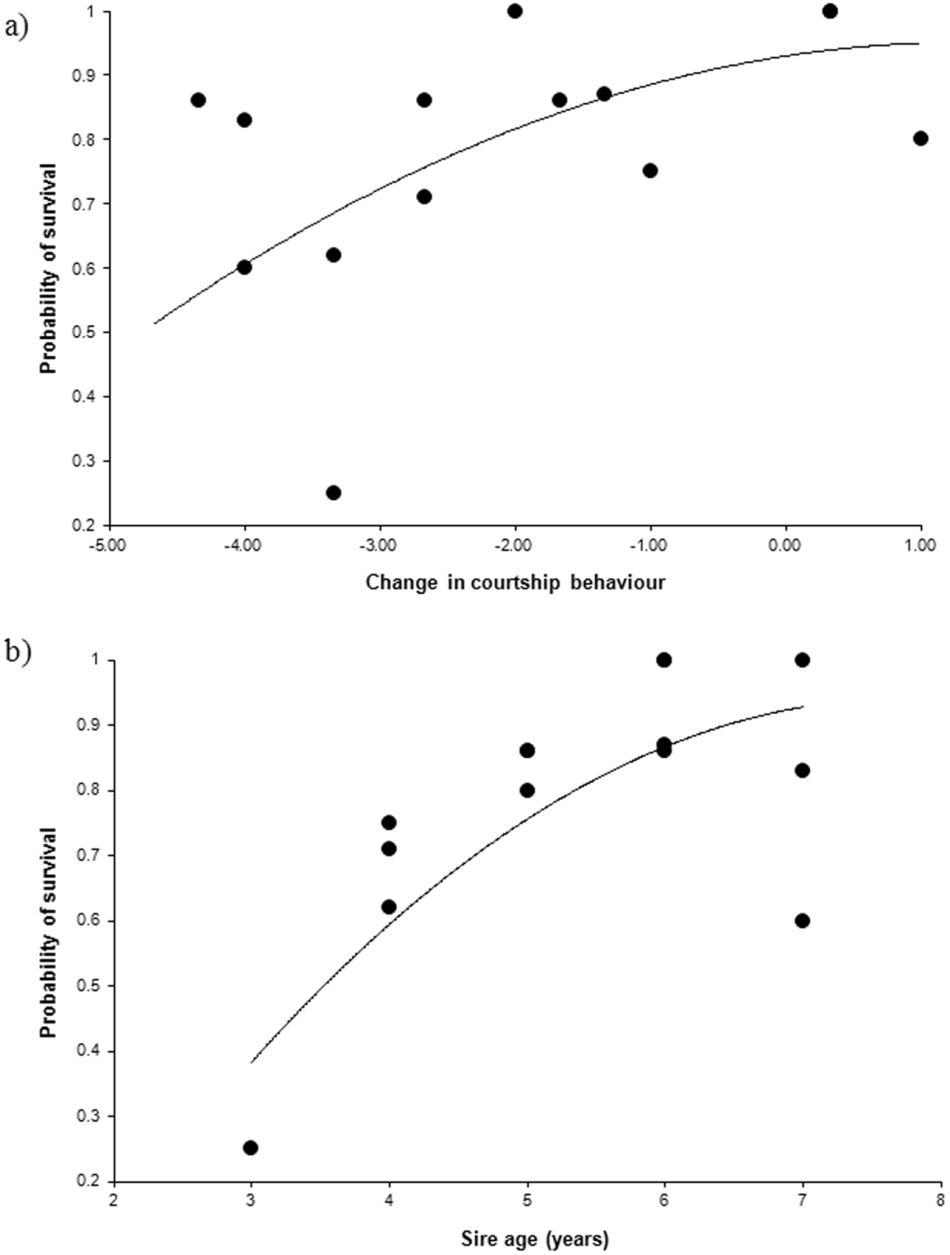
Positive correlation between offspring survival and sire traits. (a) Change in sire courtship behaviour induced by the inflammatory insult. (b) Sire age. Both plots report the percentage of offspring survival per sire with the adjustment based on the predicted values of a generalized linear mixed model where sire and dam identities were fitted as random factors.

Over the survey period, 22 birds were found dead (18.3%), 70 were recorded as being alive (58.3%), and 28 were lost (23.3%). Neither courtship display activity in 2006 nor pre-injection display in 2007 predicted offspring survival. A stepwise Cox regression model only retained paternal age as significant predictor of offspring survival (χ^2^_1_ = 7.76, p = 0.0053, hazard ratio = 0.6). The generalized linear mixed model provided the following results for the two years (display in 2006, F_1,18_ = 1.17, p = 0.2939; display in 2007 prior to the immune challenge, F_1,18_ = 1.98, p = 0.1761) (see table 1 for the full models). On the contrary, the capacity of sires to withstand the inflammatory challenge in 2007, as assessed by the difference in courtship display, significantly contributed to explain offspring survival whatever the model used (Cox regression and generalized linear mixed model, Tables 2 and 3). As expected, sires that coped with the inflammatory challenge by maintaining their display rate sired offspring with significantly better survival prospects (Fig. 2a). The age of the sire was also correlated with offspring survival in both models: the older the sire the better the survival of offspring (Fig. 2b). The age of the mother was negatively correlated with offspring survival but only in the Cox regression model (Table 3). Offspring sex, offspring body mass and age at release did not predict offspring survival in either model.

**Table 1:** Generalized linear mixed models (with a binomial error distribution and a logit link function) exploring the effect of sire courtship display in 2006 (model 1), and sire courtship display in 2007 (prior to the immune challenge) (model 2) on the survival of juvenile houbara bustards released in the wild.

*Model 1*					
Source of variation	Estimate	SE	df	F	P
Sire age	0.804	0.290	1,18	7.67	0.013
Courtship display in 2006	−0.178	0.165	1,18	1.17	0.294
Age at release	−0.048	0.025	1,18	3.66	0.072
Dam age	−0.258	0.148	1,18	3.01	0.099
Sex	(F) −1.08	1.15	1,18	0.88	0.361
Body mass at release	−0.0001	0.002	1,18	0.00	0.950

*Model 2*					
Source of variation	Estimate	SE	df	F	P
Sire age	0.846	0.289	1,18	8.58	0.009
Courtship display in 2007*	−0.178	0.165	1,18	1.98	0.176
Age at release	−0.048	0.025	1,18	3.60	0.074
Dam age	−0.273	0.146	1,18	3.50	0.078
Sex	(F) −1.25	1.52	1,18	1.17	0.293
Body mass at release	−0.0004	0.002	1,18	0.04	0.837

*Prior to the immune challenge.

The models also included sire and dam age, offspring sex, offspring body mass and age at release. Sire and dam identity were fitted as random factors.

**Table 2:** Generalized linear mixed model (with a binomial error distribution and a logit link function) exploring the effect of sire change in courtship display, sire and dam age, offspring sex, offspring body mass and age at release on the survival of juvenile houbara bustards released in the wild.

*Full model*					
Source of variation	Estimate	SE	df	F	P
Sire age	0.679	0.261	1,18	6.76	0.018
Sire change in courtship display	0.499	0.223	1,18	4.88	0.040
Age at release	−0.046	0.027	1,18	2.94	0.104
Dam age	−0.242	0.159	1,18	2.31	0.146
Sex	(F) −1.02	1.19	1,18	0.75	0.398
Body mass at release	−0.0001	0.002	1,18	0.00	0.996

*After model selection*					
Source of variation	Estimate	SE	df	F	P
Sire age	0.635	0.234	1,21	7.36	0.013
Sire change in courtship display	0.526	0.211	1,21	6.20	0.021

The model also included the sire and dam identity as random factors. The table presents the initial model and the variables that were retained in the final model.

**Table 3:** Stepwise Cox regression exploring the effect of sire change in courtship activity, sire and dam age, offspring sex, offspring body mass and age at release on the survival of juvenile houbara bustards released in the wild.

*Stepwise selection*			
Variables entered		Wald χ^2^	P
1. Sire age		7.76	0.005
2. Sire change in courtship display		12.72	0.002
3. Dam age		16.33	0.001

*Analysis of Maximum Likelihood Estimates*			
Source of variation	Hazard ratio	χ^2^	P
Sire age	0.649	6.580	0.010
Sire change in courtship display	0.610	7.022	0.008
Dam age	1.291	5.711	0.017

The table reports the order in which variables were entered into the model by the stepwise procedure and the analysis of maximum likelihood estimates for the variables that reached the 0.05 significance threshold to enter the model.

## Discussion

In this study we have shown, in an endangered lekking species, that paternal contribution can affect the probability of offspring survival in the wild. Although, natural variation in courtship display did not explain offspring survival, offspring sired by sires who were heavily affected by the experimentally induced inflammatory insult had a lower survival probability compared to offspring sired by sires who coped with the inflammatory insult. In addition to the sire ability to withstand the inflammatory challenge, sire age also contributed to the probability of offspring survival with younger sires producing the offspring with the lowest survival. These differences in survival prospects have implications both for our understanding of the evolution of female preference and the management of supportive breeding programs.

Good genes theory of sexual selection has attracted considerable attention since the idea that male phenotype can signal its genetic quality was put forward by Zahavi [2]. Several refinements were subsequently suggested by other seminal papers [3], [21]–[22]. In spite of such theoretical interest, robust empirical evidence remains sparse [23]. A major drawback associated with the studies of good genes sexual selection has been to control for environmental effects that might produce the observed benefits for the progeny, independently of paternal genes. Differential maternal investment is one of such potential environmental effects [13]. We used artificial insemination of females to largely circumvent this issue and show that paternal genes directly affected offspring survival, independently of any maternal investment in offspring. Moreover, since eggs were artificially incubated and chicks hand-raised, we are confident that the environmental conditions were identical for all offspring. Another problem usually associated with studies of viability genes inherited from the paternal lineage, is that subsequent survival is monitored under laboratory conditions. Although these conditions allow the fate of individuals to be followed with certainty, the relevance of survival measured in the absence of natural ecological conditions (with predation, competition, starvation, pathogens, etc.) is to some degree questionable. We were able to overcome these difficulties by equipping individuals with radio transmitters that allowed us to monitor the fate of each bird in their natural setting. Finally, since sires were exposed to the immune challenge when the offspring had already hatched, the effect on offspring survival cannot be imputed to LPS-induced sperm damage [20].

Although our experimental approach (artificial insemination and immune challenge) allowed us to control for a number of potential confounding factors, it also prevented us to link female choice to offspring viability. Assessing female choice is an hard task both under captive and natural conditions, and although there are several lines of evidence suggesting that females use male courtship display to choose a mate [24]–[26], we acknowledge that our study lacks a direct demonstration of the causal relationship between mate choice and offspring survival.

Contrary to several previous studies which have reported positive correlations between body mass and survival in birds [27], [28], we did not find any effect of offspring phenotype on their future survival prospect. This is an interesting result that suggests that we should be cautious when using offspring phenotype as a surrogate of quality (or fitness), especially for birds raised in captivity and experiencing benign environmental conditions prior to the release in the wild.

In addition to the possible caveats associated with the assessment of genetic benefits of mate choice, recent work has questioned the relevance of indirect sexual selection as a major force driving the evolution of female preference [29]–[31]. Here we found that the effect of paternal genes on fitness was large since the percentage of offspring per sire that were still alive at the end of the study ranged from 25% to 100%. These results, therefore, indicate that the reward of female preference can be substantial in the houbara bustard. Nevertheless, having a complete picture on the forces acting on female preference requires assessing the cost of choosiness, as well [32].

It is worthwhile to note that the effect of sire courtship display on offspring survival was significant only when sires were experimentally “forced” to face an environmental stress (the immune challenge). Natural variation in courtship display, as assessed in 2006 and 2007, did not predict the future survival prospect of wild-released young. This might be due to the benign conditions experienced by captive males in the rearing, which probably result in a reduction of among-sire variation in the expression of the sexual signal compared to the wild. Condition-dependent secondary sexual traits can be highly sensitive to environmental conditions [33] and it is therefore possible that under harsher, natural environmental conditions variation in display activity better catches variation in genetic quality than it does in captivity.

In addition to the capacity to withstand an inflammatory insult, sire age was also a good predictor of offspring survival. Again, given that juveniles experienced similar environmental conditions before their release in the field, this effect only arose from inter-individual variation in sire quality. One possible explanation for this finding could be that older sires have gone through episodes of selection such that they represent a higher quality sub-sample of the initial populations of males. However, a recent study based on a longitudinal monitoring of ejaculates across ages has shown that sperm quality increases from the age of 1 up to 4–5 years and then declines [34]. This effect does not arise because of the selective (dis)appearance of males with particular phenotypes, suggesting that this age-dependent effect has an individual (physiological) base [34]. Therefore it is tempting to speculate that age-dependent variation in sperm quality has a long lasting effect on offspring fitness well beyond the early embryo development. This hypothesis would certainly deserve to be investigated in the future. We also found that maternal age affected offspring survival, with older mothers producing offspring with the poorest survival. The range of ages covered by females was larger than for sires (2–14 years old), suggesting that aged females might have produced poor quality offspring. Although, we do not know the mechanism underlying the negative effect of maternal age on offspring survival, one possibility could be that aging mitochondria and/or poor investment into eggs reduce the survival prospect in the wild. However, this result should be treated with caution since the effect of maternal age was significant only in the Cox regression model.

In addition to provide evidence in support to the hypothesis of indirect benefits of mate choice in the houbara bustard, our results also feed the debate on the importance of mate choice for captive breeding programs [35]–[36]. One argument that has been often evoked against the practice of reproductive skew among sires in supportive breeding programs, is that this should erode genetic variation and reduce the fitness in the wild [37]–[38]. For instance, Victoria Lake cichlids maintained in a captive-breeding program have suffered a severe loss of genetic variability [39], probably due to a strong reproductive skew, with a few dominant males securing most of the copulations. Allowing females to choose a mate, or inseminating females with selected males (based on their phenotypic characteristics) can, however, improve reproductive success and offspring viability as shown here and in previous work [40]–[41].

## Materials and Methods

### Ethics statement

This work has been conducted according to relevant national and international guidelines. Birds used in the present experiment have been captive bred in the Emirates Center for Wildlife Propagation and released in the wild in agreement with Morrocan environmental policies. The study has been approved by the Ministère de l'Agriculture, Développement Rural, et des Pêches Maritimes, Direction Provinciale de l'Agriculture de Boulemane, Service Vétérinaire (N° DPA/48/285/SV). All birds have been sanitary controlled by the ONSSA (Office National de Sécurité Sanitaire des Produits Alimentaires) and all releases have been recorded by the “Haut Commissariat aux Eaux et Forêts et à la Lutte Contre la Désertification”.

### General procedure

**Figure 3:**
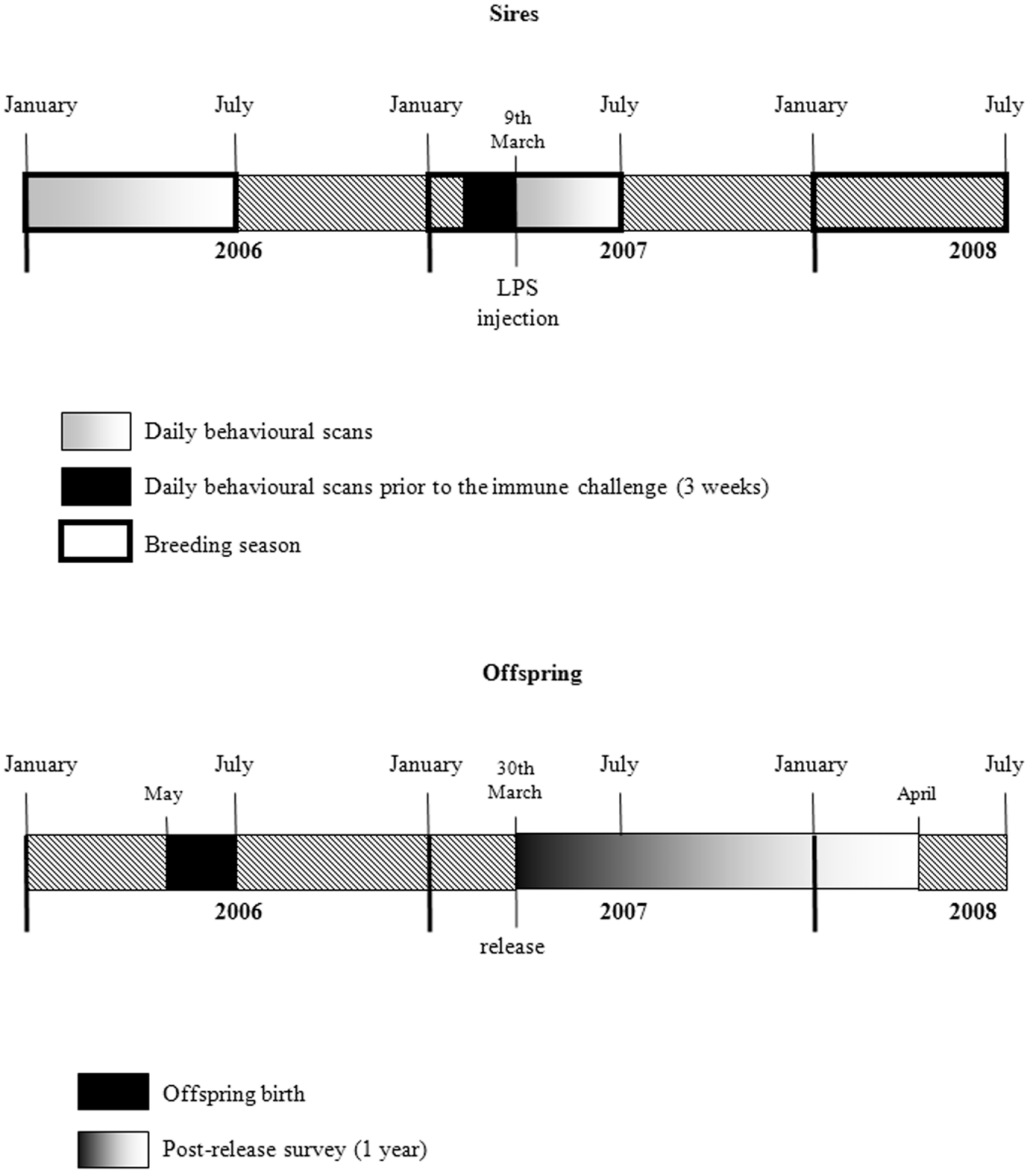
Timeline of the experiment. The figure describes the different steps of the study, for sires (daily behavioural scans, LPS injection) and for offspring (birth, release, post-release monitoring).

The study was conducted between 2006 and 2008 at the Emirates Center for Wildlife Propagation (ECWP, Missour, Morocco) using 15 adult sires aged from three to seven years and their progeny (120 offspring: eight chicks per sire) (see Fig. 3 for the timeline of the study). Each sire was mated with three to seven females in order to minimize maternal effects. A total of 83 females were used for this study. Two out of these 83 females were inseminated with two sires and the paternity (n = 5 chicks) was assigned using six polymorphic microsatellite loci (A113a-bis, A120, A210, A21, A29, D118, see Lesobre et al. [42] for further details).

Breeding birds were housed in individual outdoor cages (2 m×4 m). Food and water were provided *ad libitum*. Individuals were bred as part of an artificial insemination program and genetic management was aimed at equalizing the founders' contribution and avoiding inbreeding [42]. Sires were collected for semen every two days using the technique described in Saint Jalme et al. [43]. Briefly, sires mounted a dummy female and a glass dish was used to collect the semen by holding it under the sire cloaca during ejaculation. This technique of semen collection ensures that the quantity of semen collected closely approaches the amount ejaculated during a natural copulation. The semen was immediately transferred into a vial and used to inseminate females within 3 hours after collection. Importantly, all sires were treated using the same procedure.

Eggs laid were collected every day and transferred to an incubator where they were set in standard conditions until hatching. Overall, during the 2006 breeding season, females inseminated by the 15 sires used for this study laid 712 fertilized eggs, of these 501 hatched and 443 chicks were alive at the age of 5 months. One hundred twenty chicks were chosen as to maximize the number of dams and to have relatively homogenous ages. They were hand-reared in standard conditions until 10 days old and then housed, in groups of five unrelated birds, in outdoor aviaries (9 m×30 m) until being released into the wild (in April 2007) at the mean age (± SE) of 296 days (± 1.14) (min – max = 276–339 days).

### Courtship display

During the breeding season that lasts from January to June, male houbara bustards devote several hours per day to perform a courtship display, mainly at dawn [16]. During the breeding seasons 2006 and 2007, we performed behavioural scans of each sire three times a day (early morning, mid-day and afternoon). A ECWP staff moved around the aviaries and when a male was observed displaying at least once over the three observations, it received a score of 1; otherwise a score of 0 was assigned. Although this is a relatively coarse measure of courtship display, it is likely to catch most of the inter-individual variation. First, we found that courtship display based on three and nine daily scans are highly correlated (r = 0.71, p<0.001, n = 15 males); second, a previous study based on 28 to 32 daily scans has shown a very similar effect of LPS-injection on courtship display with a decrease in the first week following the challenge and a gradual recover to the pre-injection value [20]. Courtship activity was characterized as the number of days with courtship display per week (0–7).

### Activation of the inflammatory response

On the 9^th^ of March 2007 sires received an intramuscular injection in the thigh of 1 mg of *Escherichia coli* lipopolysaccharide (LPS, serotype 055:B5, Sigma L2880) per kg of body mass. The activation of the inflammatory response occurred when the chicks used in this study were already hatched (and almost about to be released in the wild). We choose this particular experimental design because previous work has shown that LPS injection can affect sperm and offspring quality [20], possibly confounding the relative contribution of paternal genes and of the injection on offspring survival.

### Offspring survival

The 120 juveniles (72 females and 48 males) were released in April 2007 at the Enjil plateau (Morocco). Until the release, all birds were reared in pre-release aviaries in standard conditions following the ECWP pre-release procedure (www.ecwp.org). Before the transfer to the release site, all birds were fitted with necklaces with battery-powered transmitters (10 g, 12 months battery lifespan with mortality signal, RI2B-M Holohil System Ltd). Birds were then located at least once a week during one year, using both ground and aerial radio-tracking.

### Statistical analyses

Temporal change in courtship activity, following the immune challenge, was assessed using a generalized linear mixed model (GLMM, Glimmix procedure, SAS 9.1), with time as a fixed factor and sire identity as a random factor so as to take into account the repeated nature of the data.

We aimed at assessing the effect of sire courtship display on the survival of their offspring. To do this we used two different models. First, we used a Cox regression analysis. Second, in order to take into account the family structure, we also analyzed the data with a generalized linear mixed model with a binomial distribution of errors and a logit link function. Sire and dam identities were fitted as random factors as to take into account the non-independence of sibs.

In spite of the intensive radio tracking, 28 birds were lost, possibly because of transmitter failure, or long-distance dispersal. These birds with unknown status were removed from the analyses when using the GLMM. They were, however, used in the Cox regression analysis and censored at the time when the radio signal was lost. Even though the two models differed in the total number of birds considered, the results were qualitatively very similar (see results).

Both the Cox regression and the GLMM were run twice. In the first models, we tested whether courtship display assessed in 2006 (the year when chicks hatched) and in 2007 prior to the immune challenge predicted offspring survival. These models also included offspring sex, body mass and age at release, dam and sire age. In the second model, we tested whether the injection-induced changes in courtship display (the difference between one week post- and pre-injection values) assessed in 2007 predicted offspring survival. This model also included offspring sex, body mass and age at release, dam and sire age.

Model selection of the Cox regression was based on a stepwise procedure implemented in PROC PHREG (SAS 2001) with a significance threshold fixed at 0.05. For the GLMM, model selection was achieved by removing step-by-step variables with the highest *p* values.

All the analyses were performed with SAS (v.9.1) software.
